# The SIDECAR project: S-IcD registry in European paediatriC and young Adult patients with congenital heaRt defects

**DOI:** 10.1093/europace/euac162

**Published:** 2022-09-15

**Authors:** Massimo Stefano Silvetti, Luc Bruyndonckx, Alice Maltret, Roman Gebauer, Joanna Kwiatkowska, László Környei, Sonia Albanese, Cristina Raimondo, Christian Paech, Maciej Kempa, Gábor Fésüs, Reinoud E Knops, Nico Andreas Blom, Fabrizio Drago

**Affiliations:** Paediatric Cardiology and Cardiac Arrhythmia/Syncope Unit, Department of Paediatric Cardiology and Cardiac Surgery, Bambino Gesù Children’s Hospital IRCCS, Rome, Italy; Department of Paediatric Cardiology, Amsterdam University Medical Centers, Amsterdam, The Netherlands; Laboratory of Experimental Medicine and Paediatrics, University of Antwerp, Antwerp, Belgium; Department of Paediatric Cardiology Hopital Necker-Enfants Malades, Paris, France; Hopital Marie Lannelongue-M3C, GHPSJ, Université Paris Descartes, Paris, France; Department of Paediatric Cardiology, Heart Centre Leipzig, University of Leipzig, Leipzig, Germany; Department of Paediatric Cardiology and Congenital Heart Defects, Medical University of Gdansk, Gdansk, Poland; Gottsegen National Cardiovascular Center, Budapest, Hungary; Heart Surgery Team, Department of Paediatric Cardiology and Cardiac Surgery, Bambino Gesù Children’s Hospital IRCCS, Rome, Italy; Paediatric Cardiology and Cardiac Arrhythmia/Syncope Unit, Department of Paediatric Cardiology and Cardiac Surgery, Bambino Gesù Children’s Hospital IRCCS, Rome, Italy; Department of Paediatric Cardiology Hopital Necker-Enfants Malades, Paris, France; Department of Paediatric Cardiology, Heart Centre Leipzig, University of Leipzig, Leipzig, Germany; Department of Cardiology and Electrotherapy, Medical University of Gdansk, Gdansk, Poland; Gottsegen National Cardiovascular Center, Budapest, Hungary; Department of Paediatric Cardiology, Amsterdam University Medical Centers, Amsterdam, The Netherlands; Department of Paediatrics, Antwerp University Hospital, Edegem, Belgium; Department of Paediatric Cardiology, Amsterdam University Medical Centers, Amsterdam, The Netherlands; Department of Paediatric Cardiology, Leiden University Medical Center, Leiden, The Netherlands; Paediatric Cardiology and Cardiac Arrhythmia/Syncope Unit, Department of Paediatric Cardiology and Cardiac Surgery, Bambino Gesù Children’s Hospital IRCCS, Rome, Italy

**Keywords:** Implantable cardioverter defibrillator, Subcutaneous-ICD, Paediatric age, Congenital heart disease, ICD complications

## Abstract

**Aims:**

Subcutaneous-implantable cardiac defibrillators (S-ICDs) are used increasingly to prevent sudden cardiac death in young patients. This study was set up to gain insight in the indications for S-ICD, possible complications, and their predictors and follow-up results.

**Methods and results:**

A multicentre, observational, retrospective, non-randomized, standard-of-care registry on S-ICD outcome in young patients with congenital heart diseases (CHDs), inherited arrhythmias (IAs), idiopathic ventricular fibrillation (IVF), and cardiomyopathies (CMPs). Anthropometry was registered as well as implantation technique, mid-term device-related complications, and incidence of appropriate/inappropriate shocks (IASs). Data are reported as median (interquartile range) or mean ± standard deviation. Eighty-one patients (47% CMPs, 20% CHD, 21% IVF, and 12% IA), aged 15 (14–17) years, with body mass index (BMI) 21.8 ± 3.8 kg/m^2^, underwent S-ICD implantation (primary prevention in 59%). This was performed with two-incision technique in 81% and with a subcutaneous pocket in 59%. Shock and conditional zones were programmed at 250 (200–250) and 210 (180–240) b.p.m., respectively. No intraoperative complications occurred. Follow up was 19 (6–35) months: no defibrillation failure occurred, 17% of patients received appropriate shocks, 13% of patients received IAS (supraventricular tachycardias 40%, T-wave oversensing 40%, and non-cardiac oversensing 20%). Reprogramming, proper drug therapy, and surgical revision avoided further IAS. Complications requiring surgical revision occurred in 9% of patients, with higher risks in patients with three-incision procedures [hazard ratio (HR) 4.3, 95% confidence interval (95% CI) 0.5–34, *P* = 0.038] and BMI < 20 (HR 5.1, 95% CI 1–24, *P* = 0.031).

**Conclusion:**

This multicentre European paediatric registry showed good S-ICD efficacy and safety in young patients. Newer implantation techniques and BMI > 20 showed better outcome.

## Graphical Abstract

**Graphical Abstract:**
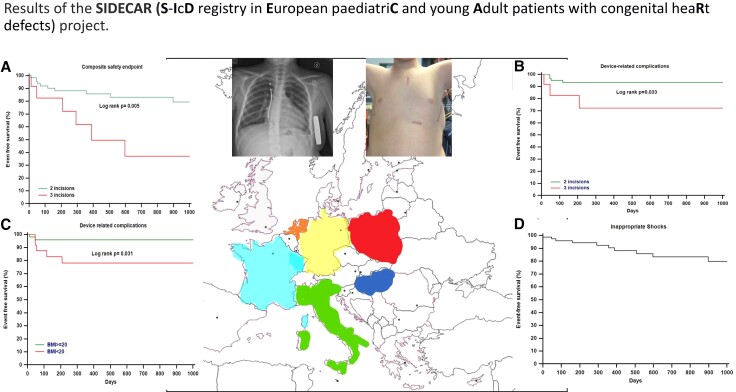

**What’s new?**

- The study provides data from a multicentre European registry on subcutaneous-implantable cardiac defibrillator outcome in paediatric and young adult patients with congenital heart disease.
- No defibrillation failure occurred over short/mid-term follow up.
- Risk factors for complications requiring surgical revision were three-incision procedures and body mass index <20 kg/m^2^.

## Introduction

Sudden cardiac death (SCD) is a rare event in young patients and most common causes are congenital heart diseases (CHDs), cardiomyopathies (CMPs), and inherited arrhythmias (IAs). Implantable cardiac defibrillator (ICD) therapy is effective for both primary and secondary prevention of SCD in adults. ICDs are used increasingly in children and adolescents, although the use of ICD in children and young patients is limited by many factors, such as the size of the devices, lead characteristics, complex anatomy, and physiologic changes during growth and physical activity. Therefore, careful patient selection is essential.^1^ The subcutaneous-ICD (S-ICD) overcomes issues related to leads and vascular/intracardiac anatomic abnormalities in growing patients,^2–6^ yet information on safety and efficacy in paediatric and adult CHD patients is limited. The aim of this study was to establish the outcome of S-ICD implantation and S-ICD therapy in paediatric and young adult patients enrolled in a European multicentre registry.

## Methods

This is a European multicentre observational, retrospective, non-randomized, standard-of-care study on outcome of S-ICD therapy in young patients, carried out by the Working Group of Cardiac Dyshrhythmias and Electrophysiology of the European Association of Paediatric and Congenital Cardiology.

We retrospectively analysed data of paediatric and young adult patients (≥18 years of age) with CHD that underwent *de novo* implantation of an S-ICD (Boston Scientific Inc.) between January 2010 and December 2020. Patients from seven European centres were included and followed up until June 2021. Paediatric patients included children (≤12 years of age) and adolescents (13–17 years). Twenty-seven patients enrolled have been included in prior studies.^5,6^ Data of patients who underwent S-ICD implantation in the participating centres were collected in a registry. The Institutional Review Board of all participating centres and local ethics committee approved the study and patients and/or their parents provided written informed consent for data storage and analysis.

Baseline assessment included the collection of demographic data and medical history, clinical examination, 12-lead electrocardiogram (ECG), and echocardiographic evaluation. Subcutaneous-ICD screening was performed by the dedicated tool in supine and sitting/standing positions. Before implantation, S-ICD eligibility was assessed through surface ECG screening in all patients. In the three centres that collected data about eligible patients, 10 out of 51 patients (19%) failed the screening test. Additional clinical information about the patients who did not fulfil the screening criteria for S-ICD implantations were not available.

The lead was positioned in a standard left or right parasternal position with the proximal end of the shocking coil located in line with the xiphoid process. Fluoroscopy was used in all patients in order to ensure positioning of the entire generator within the cardiac silhouette.

Implantation data included device and lead type, implant position, type of anaesthesia, procedure time, defibrillation testing, or any complication. Information regarding two- vs. three-incision technique and subcutaneous or intermuscular device implantation was recorded.

Follow-up data included clinical examination, 12-lead ECG, echocardiographic evaluation when required, device telemetric interrogation, and any complication.

All complications were listed and checked in each patient. Early complications were defined as happening in the first 3 months, and complications after 3 months were noted as late complications. The endpoint of this study is the outcome of S-ICD in paediatric and young adult patients, expressed as efficacy and safety. The efficacy was defined in terms of effective shocks delivered to convert ventricular fibrillation (VF) or ventricular tachycardia (VT). The composite safety endpoint comprehended all complications, and consisted of a combination of device-related complications and IASs. Device-related complications included device infection that led to generator or lead extraction, lead repositioning or replacement, and other complications related to the lead or generator requiring surgical intervention. The time to first composite endpoint was evaluated for the overall population and for patients stratified according to the implantation technique adopted and the body habitus [body mass index (BMI), <20 vs. >20 kg/m^2^].^5^

## Statistical analysis

Descriptive statistics are reported as mean ± standard deviation for normally distributed continuous variables, or median with corresponding interquartile range in the case of skewed distribution. Categorical variables are reported as percentages. Early and late complications were recorded. Survival rates were compared between groups by using Kaplan–Meier curves and a univariate Cox proportional hazards model. A *P*-value of 0.05 was considered significant for all tests. The Kaplan–Meier survival analysis was used to study freedom from adverse events. The survival curves were compared with the log-rank test. The Cox regression analysis, both univariate and multivariate, was applied to verify possible event predictors. The hazard ratio (HR), the 95% confidence interval (95% CI), and relative significance were reported for each covariate in the model. All statistical analyses were performed by ‘Means of R: a language and environment for statistical computing’ (R Foundation for Statistical Computing, Wien, Austria).

## Results

In 81 patients, an S-ICD was implanted in seven European paediatric cardiology and arrhythmias centres. There were 15 children, 51 adolescents, and 17 young adult patients. The youngest patient was 8 years old. The indications for S-ICD implantation were IA, idiopathic VF (IVF), CMP, and CHD. Five patients underwent S-ICD implantation after transvenous-ICD extraction: three due to malfunctioning leads and two for system infection. Demographics and procedure data are reported in *Table 1*. The distribution of enrolled patients according to the implantation technique was similar in all study centres.

**Table 1:** Demographics and implant procedure characteristics

	*n* = 81
Female gender	34 (42%)
Age, years	15 (IQR: 14–17), min-max: 8–31
Body mass index	21.8 ± 3.8, min-max:15.8–37
Height	167 ± 12, min-max: 132–190
Weight	62 ± 14, min-max: 30–120
Indication	
ȃPrimary prevention	48 (59%)
ȃSecondary prevention	33 (41%)
Cardiomyopathy	38 (47%)
Hypertrophic	19
Arrhythmogenic	13
Dilated	4
Non-compaction LV	2
Congenital heart disease	15 (19%)
D-TGA s/p Mustard	2
D-TGA s/p arterial switch	1
Congenitally corrected TGA and Ebstein anomaly	1
Complex congenital heart defects s/p Glenn–Fontan	4
PA + VSD	1
Coronary arteries abnormalities	3
Tetralogy of Fallot	3
Tuberous sclerosis (rhabdomyomas)	1 (1%)
Idiopathic VF	17 (21%)
Channelopathy	10 (12%)
Brugada syndrome	1
Long QT syndrome	7
CPVT	2
LV ejection fraction, %	53 ± 14
Concurrent pacemakers	4 (5%)
Previous transvenous-ICD	5 (6%)
Procedure specifications	
ȃProcedure time, min	90 (62–120)
ȃGeneral anaesthesia	74 (91%)
Device	
ȃFirst generation	15 (19%)
ȃSecond generation	66 (81%)
Three-incision technique	15 (19%)
Two-incision technique	66 (81%)
Device depth	
ȃSubcutaneous	48 (59%)
ȃIntermuscular	34 (41%)
Sternal lead position	
ȃLeft	71 (88%)
ȃRight	7 (9%)
ȃMidline	3 (3%)
Medications	
ȃBeta-blockers	59 (73%)
ȃACE inhibitors/ARBs	24 (30%)
ȃAntiarrhythmic	14 (17%)

ACE, angiotensin-converting enzyme; ARB, angiotensin II receptor blocker; CPVT, catecholaminergic polymorphic ventricular tachycardia; D-TGA, D-transposition of the great arteries; ICD, implantable cardioverter defibrillator; LV, left ventricular; PA, pulmonary atresia; s/p, status post; VSD, ventricular septal defects; VF, ventricular fibrillation.

### Subcutaneous-implantable cardiac defibrillator screening

The surface ECG screening procedure identified 1 vector in 12 patients (15% of cases), 2 vectors in 43 (53%), and 3 vectors in 26 (32%). The primary vector was suitable in 58 (72%) patients, the secondary in 72% of patients, while the alternate in 73% of patients.

### Subcutaneous-implantable cardiac defibrillator implantation

Data from the implantation procedure are reported in *Table 1*. No intraoperative complications occurred.

Defibrillation test was performed at the end of implantation procedure in all except six patients (7%) due to their clinical status (low ejection fraction). In eight patients (10%) VF was not inducible and only a 10 J test shock was given to measure impedance.

Defibrillation at shock energy of ≤65 J was successful in the remaining 67 patients (100% of VF induced). Sensing vectors were the primary in 54%, the secondary in 36%, and the alternate in 10% of the patients.

### Subcutaneous-implantable cardiac defibrillator programming

Most patients (90%) had dual-zone programming, with the shock zone programmed at a median of 250 (interquartile range 200–250) b.p.m. and the conditional zone at a median of 210 (180–240) b.p.m.

In particular, the shock zone was 250 b.p.m. in 70% of patients, 230–240 b.p.m. in 21%, and <230 in 9%. The conditional zone was >200 b.p.m. in 61% of patients, 190–200 b.p.m. in 33%, 180 b.p.m. in 6%.

Forty patients (60%) with the compatible devices were monitored at home through the LATITUDE™ system. However, we have no data about Latitude transmissions.

### Follow up

Patients were followed up for a median duration of 19 (6–35) months.

Five patients were lost to follow up. Of the remaining 76 patients, non-device-related death occurred in 2 patients (2.6%), due to cardiogenic shock and post-infective respiratory failure in 1 case each. Heart transplantation was performed in four patients (5%). Change to/addition of transvenous pacing was needed in two patients (2.8% of the study population), who did not require pacing at the time of first implantation, one for cardiac resynchronization therapy and one for addition of a transvenous pacing system to the S-ICD due to development of bradycardia.

Three patients underwent device replacement for battery depletion at a median of 5.8 years from first implantation.

### Appropriate shocks

Appropriate shocks occurred in 13 patients (17%) for VT/VF. None of the patients experienced electrical storms. Ventricular tachyarrhythmias were interrupted with the first shock in 10 patients (77%), whereas 3 patients (23%) required additional shocks: two in 2 patients and three in another. The final success of ventricular tachyarrhythmia conversion was 100%. The primary diagnosis of these patients was CMP (hypertrophic in four patients, dilated in one), IVF (in three patients), IA (long QT syndrome in two patients and catecholaminergic polimorhic VT in one patient), CHD (tetralogy of Fallot in one and status post-Fontan palliation in the other patient).

### Composite safety endpoint

Sixteen patients (21%) experienced 17 complications (details and treatments in *Table 2*): 7 device-related complications and 10 IASs. Two complications (lead tip wound erosion and IAS due to noise oversensing) were detected at the same time in one patient. Early complications occurred in seven patients (44%) and late complications in nine patients (56%). Results from the regression analysis of variables associated with the composite safety endpoint are reported in *Table 3*.

**Table 2:** Clinical events occurred during follow up

Events	Number of patients	Early/late	Management	Outcome
Non-device-related death	2	0/2		
Heart transplant	4	0/4		
Need for CRT	1	0/1	Transvenous pacemaker	Solved
Bradycardia	1	0/1	Transvenous pacemaker	Solved
Battery depletion	3	0/3	Device replacement	Solved
Components of the composite safety endpoint				
Lead tip erosion	1	1/0	Surgical revision	Solved
	2	1/1	Repeated surgical revisions	Explanted
Local pocket infection	1	1/0	Explant	Explanted
	1	1/0	Pocket revised	Solved
Impaired wound healing	1	1/0	Local disinfection	Solved
Lead dislodgment	1	0/1	Lead repositioning	Solved
Inappropriate shock (total)	10			
T-wave oversensing	4	0/4	Device reprogramming	Solved
Non-cardiac oversensing	1	1/0	Entrapped subcutaneous air: no action	Solved
	1	1/0	Revision due to lead tip erosion	Solved
Atrial fibrillation or supraventricular tachycardia	2	1/1	Device reprogramming	Solved
	2	0/2	Change in medications	Solved

Early complications were defined as happening in the first 3 months, and complications after 3 months were noted as late complications.

CRT, cardiac resynchronization therapy.

**Table 3:** Regression analysis of clinical and implantation variables associated with the composite safety endpoint

	Univariate analysis		
	HR	95% CI	*P*-value
Gender	2.269	0.783–6.572	0.133
Age	0.997	0.899–1.106	0.961
Secondary prevention	0.724	0.264–1.987	0.532
Body mass index	0.973	0.848–1.117	0.700
Channelopathy	1.382	0.395–4.831	0.614
Cardiomyopathy	0.756	0.276–2.073	0.588
Congenital heart disease	1.212	0.392–3.744	0.739
Ejection fraction	1.002	0.969–1.038	0.886
Concurrent pacemaker	2.053	0.469–8.989	0.342
Previous transvenous-ICD	0.999	0.133–7.536	0.999
Device generation	1.618	0.562–4.656	0.375
Three-incision technique	3.896	1.411–10.759	0.009
Subcutaneous pocket	1.389	0.448–4.306	0.572
Dual zone	0.320	0.072–1.416	0.135
Primary vector sensing	1.046	0.364–3.002	0.934

95% CI, confidence interval; HR, hazard ratio; ICD, implantable cardioverter defibrillator.

Inappropriate shocks occurred in 10 patients (13%). Three events occurred early (within 3 months) (*Table 2*). The primary diagnosis of these patients was: CHD (three patients, with tetralogy of Fallot, status post-Glenn and status post-Fontan palliation, respectively), IVF (three patients), IA (two patients with long QT syndrome), CMP (two patients, one with hypertrophic and one with dilated CMP).

T-wave oversensing occurred in four patients: two with S-ICD A219, one with S-ICD A209, one with S-ICD 1010. In the S-ICD devices A209 and 1010, the SMART Pass, a novel sensing methodology was not available.^7^ In the devices (A219) that include the SMART Pass option, it was disabled in one patient and enabled in the other. Device reprogramming (higher limits of fibrillation detection, change of detection vector) and surgical revision were able to resolve the oversensing (*Table 2*).

Entrapped air around the lead that caused non-cardiac oversensing (one case with long QT syndrome) resolved spontaneously.

**Figure 1:**
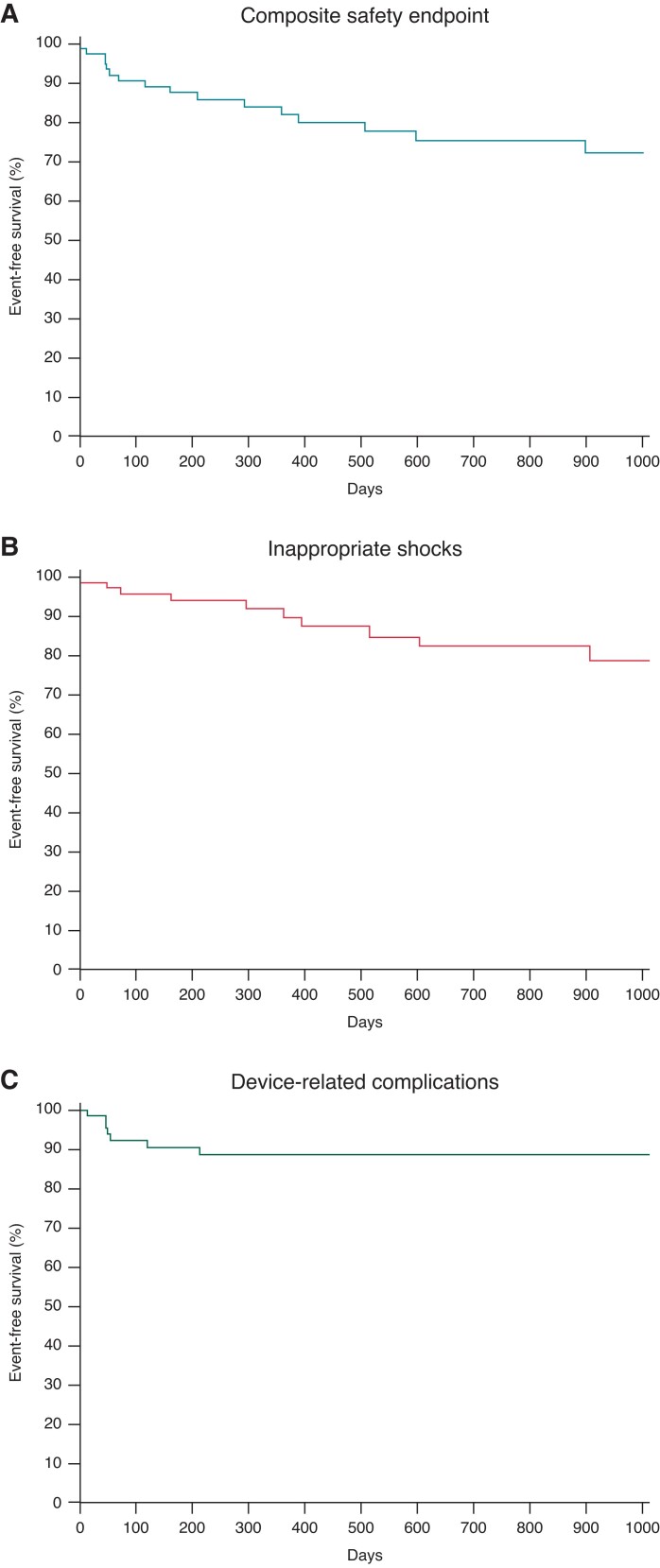
Time to first event curves for the composite safety endpoint and its components. (*A*) Composite safety endpoint. (*B*) Inappropriate shocks. (*C*) Device-related complications.

**Figure 2:**
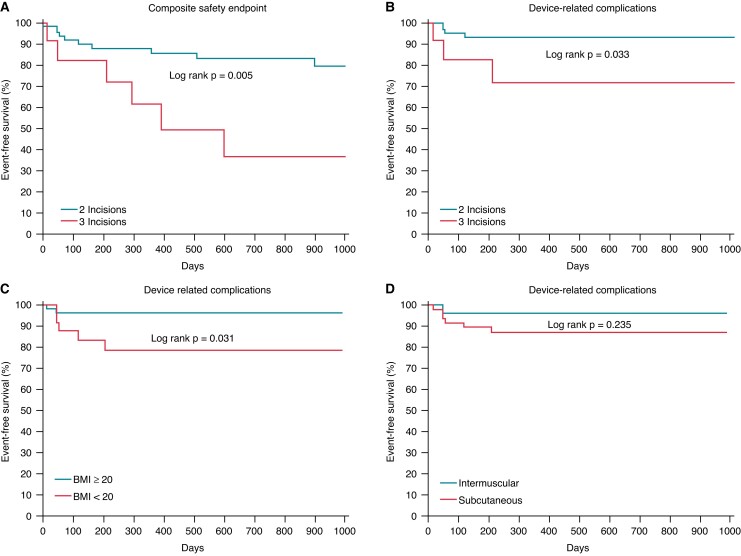
Time to first event curves for the composite safety enpoint. (*A*) in patients who had undergone implantation via the three-incision technique vs. the two-incision technique; (*B*), time to first event curves for device-related complications (three-incision technique vs. the two-incision technique); (*C*), time to first event curves for device-related complications in patients with BMI < 20 vs. BMI ≥ 20; (*D*), time to first event curves for device-related complications in patients with intermuscular vs. subcutaneous pocket.

Surgical/infective complications requiring system revision (*Table 2*) occurred in 7 patients (9%), 5 (71%) in the first 3 months of follow up, and 2 (29%) thereafter. Univariate analysis showed a significant association with BMI < 20 kg/m^2^ (HR 5.052, 95% CI 0.998–25.822, *P* = 0.05) and the three-incision technique (HR 4.399, 95% CI 0.991–19.526, *P* = 0.05). These variables were both predicting factors for complications requiring system revision at multivariate analysis: BMI < 20 (HR 7.302, 95% CI 1.359–39.228, *P* = 0.021) and the three-incision technique (HR 6.969, 95% CI 1.49–32.437, *P* = 0.014). There seemed to be a trend towards less complications in intermuscular devices in comparison with subcutaneous devices, yet this trend was non-significant (*P* = 0.235). *Figure 1* shows the Kaplan–Meier survival curves for the composite safety endpoint and its single components (device-related complications and IASs), in the overall population. *Figure 2* shows the Kaplan–Meier survival curves of patients with two- or three-incision technique, BMI > or <20, and intermuscular or subcutaneous device implantation.

## Discussion

This European multicentre study demonstrates the results of S-ICD implantation in a relatively large cohort of young patients, of whom almost three quarters were under the age of 18 years.

Subcutaneous-ICDs were implanted in patients ≥8 years of age who met screening criteria and did not require bradycardia and antitachycardia pacing. Five per cent of these patients already had a pacemaker for bradycardia pacing. The indication for ICD therapy was primary prevention in the majority of patients.

The newer two-incision technique was used in this young patient population with good cosmetic results.^5^ There was a low rate of surgical-related complications and no intraoperative complications occurred. Body dimensions (BMI) and surgical techniques were significantly associated with the occurrence of complications during follow up. Patients who underwent a three-incision technique showed significantly more complications. Complications were not related to the recent advisory affecting the 3501-model lead and not to intermuscular device location, despite a non-significant trend towards less complication in intermuscular devices in comparison with subcutaneous devices.

Defibrillation failures were not observed, and most patients received effective defibrillation by only one or two of the five available shocks. It is noteworthy that defibrillation testing was performed in most patients in our series and was 100% successful and that all patients with clinically significant ventricular arrhythmias were successfully defibrillated. New data in adult patients suggest that defibrillation testing after S-ICD implantation can be avoided comparable with current practice for transvenous-ICD implantations.^8^ The rate of IASs was 13% and similar to those reported in previous studies on S-ICDs in paediatric patients^3,6^ and in line with those reported for transvenous-ICD.^1^ In general, the occurrence of IASs can be reduced by: (i) the use of dual-zone programming, i.e. the conditional zone (with the discrimination algorithms active) and the shock zone, with high rate limits for VT/VF; (ii) technical improvements such as the new algorithms SMART Pass^7^ and more accurate screening methods that may reduce oversensing and IASs; and (iii) the remote follow up with Latitude system, that can lead to an early diagnosis of malfunctions and complications.

### Comparison with paediatric and adult implantable cardiac defibrillator literature data

Over the last decade, the S-ICD has become a new and interesting tool for the treatment of ventricular arrhythmias.

Common indications for S-ICD are young age, primary prevention, impaired vascular access, previous infection, and high infection risk. There are several contraindications such as antibradycardia/antitachycardia pacing indications and failed screening.^9^

Most patients of this study who received an S-ICD had a CMP (47%), of which most patients had a hypertrophic CMP. The percentage of patients with a CMP seems comparable with data from a recent study from von Alvensleben *et al*.^10^ on S-ICD in children, yet higher than older studies such as Bettin *et al*.^11^ (16%) and Griksaitis *et al*.^4^ (21%). This might reflect a recent liberalization of the indications of S-ICD implantation, especially in hypertrophic CMP.

Some retrospective studies have evaluated S-ICD results in young patients. An overview of them is reported in *Table 4*. They included single- and multicentre studies.^2,3,5,6,10–12^ Mainly adolescent patients, with BMI around 23 kg/m^2^, were mostly implanted for primary prevention of CMP, IA, CHD, and other diseases. The three-incision/two-incision implantation procedures were almost equally distributed, and the devices were located in an intermuscular pocket location in one-fifth of patients.

**Table 4:** Overview of paediatric studies on subcutaneous-implantable cardiac defibrillators

Author (reference)	Centre, years	No. pts	Age (years) Median (range)	Age ≤18, *n*	Weight (kg)	BMI (kg/m^2^)	CHD (%)	CMP (%)	IA (%)	Others (%)	Primary prevention (%)	3-incision (%)	2-incision (%)	Inter- muscular, (%)	DFT (%)	Shock zone (b.p.m.)	Conditional zone (b.p.m.)	Appropriate shocks (%)	Inappropriate shocks (%)	Surgical complications (%)	FU (year)
Jarman *et al*.^2^	Single, 2010–11	16	20 10–48	7	60	na	25	0	75	0	na	100	0	0	100	220 190–250	190 180–210	19	25	19	9
Pettit *et al*.^3^	Dual, 2007–11	9	15 10–18	9	54	na	na	na	na	na	56	100	0	0	100	na	na	22	11	0	2
Bettin *et al*.^11^	Single, 2011–16	31	20 13–26	na	71	23	7	32	58	3	42	na	na	na	na	240 220–250	220 190–230	26	16	3	2
Quast *et al*.^6^	Single, 2002–15	35	19 13–20	na	74	na	3	28	69	na	54	26	74	3	94	250 240–250	200 200–200	27	14	25	3.5
Silvetti *et al*.^5^	Single, 2013–17	15	15 10–31	10	60	23	40	53	7	0	93	40	60	10	73	250 210–250	220 180–230	14	7	27	1
von Alvensleben *et al*.^10^	Multi, 2010–18	115	17 5–19	na	71	24	32	40	13	16	55	53	47	18	85	na	na	11	16	4	2.5
Sarubbi *et al*.^12^	Single, 2014–20	21	14 8–18	21	59	23	19	24	43	14	33	19	81	86	76	250	210 200–220	9	19	9	3.5
Total/mean		242	17	107	64	23	21	29	44	6	55	56	44	20	88	242	208	18	15	12	3
SIDECAR study^a^	Multi, 2010–20	81	15 8–31	64	62	22	20	47	12	21	59	19	81	41	83	250 200–250	210 180–240	17	13	9	1.5

BMI, body mass index; CHD, congenital heart disease; CMPs, cardiomyopathies; DFT, defibrillation testing; FU, follow up; IA, inherited arrhythmias; *n*, number

27 patients were already reported in previous papers^5,6^ but with shorter follow up.

The results of these studies clearly show the efficacy and safety of S-ICD in the treatment of malignant ventricular arrhythmias in young patients and are comparable with those of this multicentre study.

Studies showed that appropriate shocks were delivered in 9–27% of patients (*Table 4*). In adult subjects, the S-ICD was shown to be equally effective to transvenous-ICD in the termination of malignant ventricular arrhythmias.^13^ To our knowledge, only one patient in previous paediatric studies experienced unexplained failure to successfully convert ventricular tachyarrhythmias.^10^

Subcutaneous-ICD IASs occurred mainly due to T-wave oversensing and myopotentials. More recent Emblem ICDs have a SMART Pass option that allows to successfully reduce oversensing of T-waves with unchanged R-wave registration.^7^ It has been shown that acquiring a template during exercise testing and reprogramming, the therapy zones and/or the sensing vector accordingly may reduce IASs.^14^ However, 70% of IASs occurred late (i.e. >3 months after implantation, *Figure 1B*) which could be caused by changes of QRS/T-wave signals related to pubertal growth and to the evolution of some diseases, for example, the CMPs, underscoring the need for close follow up of these patients. Paediatric S-ICD literature data report a relatively high rate of IASs from 7 to 25% (*Table 4*). This is high when compared with the rate of 2.5% in the adult patients from the UNTOUCHED trial,^15^ where predictors of IASs were atrial fibrillation history and two-incision implantation procedure. This latter finding has been related to distal lead malposition, due to its migration in absence of a suture or to an improper placement in absence of direct visualization by the implanting physician. However, another study^16^ demonstrated that the two techniques did not show significant differences in terms of mortality rates, shock efficacy, defibrillation testing, IASs, and surgical wound complications, but the two-incision technique shortened procedural times.

On the contrary, the current study found that the three-incision technique was the only predicting factor for complications requiring system revision. Indeed, one IAS episode was caused by wound erosion at the upper sternum with lead tip exposure causing non-cardiac oversensing. Other investigators^11^ found that younger age was predictor of S-ICD IASs.

Non-cardiac oversensing caused another IAS early after S-ICD implantation (first postoperative day), due to entrapped subcutaneous air at the incision of the lead dipole (lower sternum). This complication can be prevented by the routine use of a chest X-ray after the procedure to exclude the presence of air bubbles along the lead or device before the activation of the S-ICD therapies. In this patient, air bubbles were spontaneously reabsorbed.

On the basis of the current study and other previous studies,^5,10,12^ the S-ICD implant procedure in paediatric patients seems to be very safe without significant intraoperative complications.

Over follow up, instead, some authors reported surgical-related complications in 0–27% (in average, roughly, 12%) of paediatric S-ICD (*Table 4*). In the PRAETORIAN study, conducted on a different population of adult patients, these complications occurred less frequently in S-ICD than in transvenous-ICD.^13^ In the current study, no lead malfunction occurred, and the three-incision technique implantation procedure was a risk factor for complications requiring surgical revision together with a BMI < 20. This finding is clearly related to the dimensions of the device: too large for children and adequate for adolescents.

Device dimensions could become smaller in the future. The results of trials of defibrillation testing at lower energy level may lead to safe defibrillation at energy <80 J and, therefore, to the production of smaller devices.

In addition, the need of ECG screening and the lack of antitachycardia/bradycardia pacing are limitations to S-ICD use. Electrocardiogram screening is a main limiting factor in the paediatric and young adult population as S-ICD ineligibility occurred in 20% of young patients,^5^ and in 17% of adult patients with CHD.^17^ Moreover, 2.8% of patients from this study shifted to transvenous pacing for bradycardia. The IDE and EFFORTLESS registries described 0.4% of adult patients who required S-ICD removal and ventricular pacing.^18^ However, these disadvantages are overcome by the advantages of S-ICD: the implantation procedure is simpler, but more extensive surgically compared with the transvenous device (a learning curve is required). Device programming is simpler with S-ICD, although this does not necessarily represent an advantage, as the more options that are available with transvenous-ICD may translate into better patient management. Further advantages are the absence of endocardial or epicardial devices, as well as easier and lower-risk extraction procedure. Finally, shocks delivered by an S-ICD seem less harmful to the myocardium, since the troponin rise seen in patients who received a transvenous shock is not observed after a subcutaneously delivered shock.^9^

Current study and literature data show higher complication rates in the paediatric S-ICD population than in adults. Similarly, complications (17–39%) and IASs (20–30%) in children with transvenous-ICD are much more frequent than in adults.^1,19,20^ Procedural complications and IASs seem to be less with the S-ICD, but a direct comparison with transvenous-ICD has not been made. In addition, the follow-up period of most patients with S-ICD is shorter than with transvenous-ICD. Further studies are warranted to allow a comparison with transvenous-ICD and longer follow up after S-ICD implantation.

### Limitations

This is a retrospective study. The study cohort is relatively large for paediatric studies, but still underpowered for more accurate statistical evaluation, for example when considering patient subgroups’ analysis (such as intermuscular device location, etc.). Follow up may not be long enough to show all complications. Screening test data were not available in some centres. Likewise, data about Latitude transmissions were not available. However, S-ICD Latitude transmissions are not automatic and require patient/family actions.

## Conclusions

This multicentre European paediatric registry showed good S-ICD efficacy and safety in paediatric and young adult patients. Defibrillation failures did not occur. The two-incision implantation procedure and patients with BMI > 20 showed better outcome.

## Data Availability

The data underlying this article will be shared on reasonable request to the corresponding author.
